# Association of American Medical Editors

**Published:** 1888-06

**Authors:** 


### THE ASSOCIATION OF AMERICAN MEDICAL EDITORS.

The annual meeting of this Association was held May 7, in Cincinnati. The President, Dr. W. Porter, of St. Louis, delivered the annual address, in which he said:

"Two things have been clearly demonstarted during the year that has gone: First, the power of the medical press; second, the value of unity."

After advocating co-operation on the part of medical editors for the advancement of medical journalism, he said:

"A strong association can accomplish much where individual effort may be futile. There are many questions which demand attention. The further advancement of our State and national associations, legal control of quackery, the international copyright, the questions proposed for discussion to-night, the upholding of our best schools and journals, and the exposure of poor ones—these are some of the matters of vital importance which call for harmonious and well organized action by the medical press of the land.

"I would oppose limitation of the utmost freedom of thought or speech."

At the close of the address, the question as to the effect of the publication of trade journals on the regular medical journals was very thoroughly discussed by the Association; the almost unanimous voice of the body being that the effect on medical journalistic literature is bad, and in every way demoralizing to the medical profession at large, and depresses and lowers the plane of legitimate journalism.

After the adoption of a constitution and by-laws for the government of the Association, officers for the ensuing year were elected, and the society adjourned.

The next annual meeting will be held in Newport, Rhode Island.

